# Clinical Considerations of Amikacin Pharmacotherapy in Adults—A Narrative Review with Focus on Safety and TDM

**DOI:** 10.3390/antibiotics15060534

**Published:** 2026-05-24

**Authors:** Daniel Orzechowski, Aleksandra Mroczkowska, Adrian Bryła, Anna Rapacz

**Affiliations:** 1 Department of Pharmacodynamics, Faculty of Pharmacy, Jagiellonian University Medical College, 30-688 Krakow, Poland; 2 Department of Clinical Pharmacy, Ludwik Rydygier Memorial Specialized Hospital, 31-826 Krakow, Poland; aleksandra.mroczkowska@rydygierkrakow3.pl (A.M.); adrian.bryla@uj.edu.pl (A.B.); 3 Department of Clinical Pharmacology, Chair of Pharmacology, Faculty of Medicine, Jagiellonian University Medical College, 30-688 Krakow, Poland

**Keywords:** amikacin, therapeutic drug monitoring, nephrotoxicity, ototoxicity

## Abstract

**Background:** Amikacin remains a key agent in the treatment of severe and complicated infections due to its bactericidal activity and low risk of Clostridioides difficile infection. It retains activity against most aerobic Gram-negative bacteria, including multidrug-resistant Enterobacterales and Pseudomonas. However, its use is limited by nephrotoxicity and ototoxicity. **Methods:** This narrative review evaluates clinical indications, pharmacokinetic and pharmacodynamic properties, dosing strategies, therapeutic drug monitoring (TDM), and safety profile of amikacin in adult patients based on 56 selected publications. A total of 24 articles were identified through database searches (PubMed and Embase), complemented by 32 additional sources to provide clinical and pharmacological context. **Results:** Available evidence demonstrates considerable uncertainty regarding the comparative effectiveness of different monitoring strategies. Lower trough concentrations are generally associated with reduced nephrotoxicity; however, an optimal safety threshold has not been clearly established. Guideline-recommended targets vary substantially and are supported by low-quality evidence. Amikacin pharmacokinetics, tissue penetration and toxicity are influenced by patient-specific factors, including critical illness, renal function variability, and concomitant nephrotoxic therapy, particularly vancomycin. Ototoxicity remains an additional clinically relevant concern. **Conclusions:** Current evidence suggests that uniform dosing and monitoring paradigms are insufficient. Patient-tailored strategies integrating TDM and mitigation of modifiable risk factors are required. Prospective studies comparing monitoring regimens are needed to optimize the safe clinical use of amikacin and inform future guideline development.

## 1. Introduction

Amikacin is an aminoglycoside antibiotic with concentration-dependent bactericidal activity and clinically relevant post-antibiotic effect (PAE). After active transport into the bacterial cell, amikacin binds irreversibly to the 30S ribosomal subunit (mainly 16S rRNA), leading to impaired initiation of mRNA translation, its misreading and premature termination of protein synthesis. The resultant aberrant proteins increase cell membrane permeability, which further enhances intracellular drug intake and potentiates the bacterial killing effect. The duration of the PAE depends on the pathogen and the achieved peak concentration, which supports extended interval and once-daily dosing (ODD) strategies in routine clinical practice [1].

### 1.1. Spectrum of Activity

The antimicrobial spectrum of amikacin is primarily directed against Gram-negative bacteria, including clinically important Enterobacterales such as *Klebsiella* spp., *Escherichia coli* spp., *Proteus* spp., *Serratia* spp., *Citrobacter* spp., and *Providencia* spp., some of which are inducible AmpC producers of β-lactamase with limited therapeutic options. Amikacin also covers the spectrum of non-fermenting Gram-negative pathogens such as *Pseudomonas aeruginosa* and *Acinetobacter* spp., and shows in vitro activity against selected Gram-positive organisms (e.g., *Staphylococcus aureus*). Although resistance mechanisms exist (ribosomal target modifications, decreased permeability, efflux pumps and aminoglycoside-modifying enzymes), amikacin is considered to have a comparatively lower propensity for resistance selection than gentamycin. A notable feature is activity against some nontuberculous mycobacteria (e.g., *Mycobacterium avium complex*, *M. chelonae* and *M. fortuitum*) [2,3], which has supported inhaled use in tuberculosis; however, this indication is beyond the scope of this manuscript due to different drug exposure and adverse effects associated with the route of administration. Relating the above spectrum to epidemiological data published by The Lancet [4], it should be underscored that amikacin remains active against five of the pathogens responsible for the highest number of deaths in the largest global analysis to date of causes of mortality associated with bacterial infections.

### 1.2. Clinical Indications

Registration indications for amikacin use are broad and include hospital-acquired pneumonia, intra-abdominal (including postoperative) infections, infective endocarditis, urinary tract infections (UTIs), skin and soft tissue infections, and infections in neutropenic patients. In clinical daily practice, its use is significantly limited by pharmacokinetic limitations (poor penetration into non-hydrophilic tissues), local physicochemical conditions within infectious foci (e.g., low pH and hypoxia) and the risk of toxicity. As a result amikacin is most commonly used for severe or complicated urinary tract infections, where it can be used in monotherapy. Another key clinical application is use as part of combination therapy (especially often with beta-lactam antibiotics due to a chance of synergism) for severe systemic infections, particularly when multidrug-resistant strains are suspected. This highlights the important role of amikacin in intensive care units (ICUs) and hematology, where its rapid onset of action and potent bactericidal activity confer substantial value despite the associated risk of toxicity. The drug remains clinically relevant for infections caused by carbapenemase-producing Enterobacterales after microbiological confirmation of susceptibility in UTIs, and synergy has been reported with novel beta-lactam/beta-lactamase inhibitors such as ceftazidime–avibactam [5,6,7].

The aim of this narrative review is to synthesize clinically oriented evidence on amikacin therapy with an emphasis on dosing strategies, therapeutic drug monitoring and safety. Particular focus is placed on the risk of nephrotoxicity and on evaluations of different TDM regimens.

## 2. Results

### 2.1. Highlights

- Integration of pharmacokinetic data with its contribution to clinical practice.
- A discussion of the clinical use of amikacin in the context of current guidelines.
- An analysis of TDM—guidelines with particular emphasis on safety and the risk of nephrotoxicity.

### 2.2. Pharmacokinetic and Pharmacodynamic Properties in Clinical Practice

Amikacin is very poorly absorbed from the gastrointestinal tract; therefore, it is administered parenterally, primarily via intravenous infusion or less commonly by intramuscular injection. In patients with preserved renal function and normal creatinine clearance, the elimination half-life of amikacin is relatively short (approximately 1.9–2.6 h). The drug is eliminated unchanged predominantly via renal glomerular filtration, with 95% of an intramuscular dose excreted within 24 h. Amikacin is a highly hydrophilic drug that exhibits negligible plasma protein binding [8], which makes pharmacokinetic drug–drug interactions due to protein binding unlikely. The pulmonary penetration of the drug is generally considered limited. Drug concentrations in the epithelial lining fluid (ELF)—a key compartment in the pathophysiology and treatment of ventilator-associated pneumonia (VAP)—are typically suboptimal; therefore, amikacin is not recommended as monotherapy for VAP. In a study of critically ill patients receiving amikacin at the dose of 20 mg/kg, a mean peak plasma concentration of approximately 60 mg/L was associated with an ELF concentration of only about 6.3 mg/L. Peak concentrations are typically measured 30 min after the end of intravenous infusion, while trough levels are obtained immediately prior to the next dose. This limited pulmonary exposure is likely related, at least in part, to altered pharmacokinetics observed in patients in intensive care units [9]. Amikacin concentrations in ELF vary in different studies, and the drug may be effective only against less resistant bacteria [10]. Importantly, the timing of ELF sampling relative to drug administration in pneumonia studies is often heterogeneous or insufficiently reported, which represents a methodological limitation and may affect the interpretation of pulmonary exposure data. Despite these limitations, amikacin is used in ventilator-associated pneumonia exclusively as part of combination therapy.

Amikacin’s clinical efficacy is also limited in the treatment of intra-abdominal infections. In peritoneal fluid, amikacin usually reaches 50–100% of the serum concentration, but its actual bactericidal activity is limited in comparison with in vitro studies [11]. Aminoglycosides require an alkaline environment (optimal pH 7.8–8.0) to effectively penetrate bacterial cell membranes. Within abscesses and inflammatory foci in the abdominal cavity, the local pH often drops below 6.0. This is considerably lower than both the optimal pH for their activity and the pH generally reported for the epithelial lining fluid. In such conditions, there is a significant inhibition of amikacin transport into bacterial cells. Furthermore, amikacin’s mechanism of action depends on the presence of oxygen, since its uptake by bacteria is oxygen-dependent. Consequently, in anaerobic environments—such as those characteristic of intra-abdominal abscesses—the clinical efficacy of amikacin remains markedly limited as MIC values increase 10–80-fold [12,13]. As a consequence, in one of the few studies by Carrie [14] involving patients with a wide range of complex abdominal injuries and conditions, very high doses (25–35 mg/kg) were administered, despite the substantial risk of toxicity associated with such dosing. This concern is reflected in current clinical guidelines, which do not recommend amikacin for the treatment of either hospital- or community-acquired intra-abdominal infections.

In some clinical scenarios, however, patients with intra-abdominal infection may benefit from the use of amikacin. Such situations may occur in cases of concomitant bacteremia, particularly when amikacin is used as part of combination therapy with agents that achieve adequate penetration into the primary source of infection and provide an appropriate antimicrobial spectrum coverage.

Despite being formally indicated for use, amikacin is not recommended in the treatment of infective endocarditis, as confirmed by European guidelines [15]. This is due to the availability of more effective antimicrobial agents and the prolonged duration of therapy required for endocarditis, which would be associated with an unacceptably high risk of toxicity. The role of other aminoglycoside antibiotics has been greatly reduced.

Penetration into skin and soft tissues is moderate but generally sufficient for susceptible bacterial strains, with the exception of necrosis. Soft tissue levels reach approximately 30–60% of the corresponding serum concentrations and penetration in muscle, and subcutaneous tissue remains at an acceptable level even in critically ill patients [13,16]. This can be explained by the fact that, as a hydrophilic drug, it diffuses easily into the interstitial spaces and shows negligible intracellular accumulation and poor penetration into adipose tissue. However, it should be emphasized that in infections with impaired perfusion and significant tissue acidosis—such as diabetic foot infections—the clinical efficacy of amikacin may be significantly limited, for reasons analogous to those mentioned for intra-abdominal infections. Considering serum concentrations alone, one might get the impression that the drug is effective against bacteria with MIC = 8 mg/L. However, taking into account an approximate tissue penetration of 40%, the actual concentration in soft tissue would only be ~3 mg/L, which is sub-therapeutic and, even in combination with the post-antibiotic effect, may not yield clinically acceptable results.

In clinical practice, the most common use of amikacin is the treatment of severe, complicated and recurrent urinary tract infections. The pharmacokinetic properties of amikacin come in favor of its efficacy. While peak serum concentration is typically 20–60 mg/L depending on the regimen, the drug’s levels in urine reach 100-fold the plasma concentrations within the first hour [17]. The study of patients with UTI caused by extended-spectrum beta-lactamase producers showed 97.2% clinical success of amikacin therapy, indicating that amikacin is a safe alternative to carbapenems [18]. Furthermore, an analysis of data from over 13,800 patients showed that a single dose of an aminoglycoside (including amikacin) resulted in a 94.5% microbiological cure rate [17].

Infectious Diseases Society of America (IDSA) guidelines recommend the use of amikacin in guidance on the treatment of UTIs caused by resistant Gram-negative infections [6].

Certain pharmacodynamic properties of amikacin may support its use in selected clinical scenarios, even when other agents are recommended as first-line therapy by current guidelines. Amikacin is not metabolized by p450 cytochrome and therefore it does not participate in hepatic drug–drug interactions of the first-phase metabolism. In patients at risk of severe adverse events due to drug–drug interactions, amikacin can also be an important therapeutic alternative. Additional factors may also influence the clinical decision-making in favor of amikacin. Notably, aminoglycosides are associated with a relatively low risk of causing *Clostridioides difficile* infection and pseudomembranous colitis [19]. Regarding the high mortality associated with this complication, especially in patients with recurrent disease, amikacin may be considered as an option even if it would not otherwise be the first-choice agent. Furthermore, the use of aminoglycosides, including amikacin, is associated with significantly lower costs compared with the novel combinations of beta-lactam/beta-lactamase inhibitors such as ceftazidime–avibactam. As a result, amikacin may represent a valuable therapeutic option, particularly in low-resource and low-income settings.

### 2.3. Safety Profile and Adverse Effects

Amikacin is a drug with a narrow therapeutic index. Its characteristic adverse effect—similar to other aminoglycosides—is nephrotoxicity. The drug accumulates in renal cortical cells and damages proximal tubules, leading to loss of their function due to acute tubular necrosis. The risk of kidney injury increases with the duration of therapy and is particularly elevated in elderly patients and in those with concomitant risk factors such as chronic kidney disease. Concentration, which is a biomarker of amikacin accumulation and its toxicity, tends to increase with age. Steffens et al. stated that elderly patients had higher C_max_ target attainment, while the same groups had lower C_min_ target attainment [20]. Aquino et al. suggested that the impact of aging is greater than that of chemotherapy [21]. Nakayama et al. stated that systemic clearance of amikacin was significantly lower in the cachectic patient group; however, no difference in the incidence of acute kidney injury was observed [22]. Dupont [23] and Roger [24] described the pharmacokinetics of amikacin in critically ill patient populations requiring various types of renal replacement therapy and proposed different dosing regimens ranging from 15 to 35 mg/kg, most of which required extended dosing intervals exceeding 36 h.

Unlike patient-related risk factors, which are largely non-modifiable, pharmacotherapy represents a modifiable contributor to the risk of amikacin-induced nephrotoxicity. The concomitant administration of other nephrotoxic agents, such as widely prescribed non-steroidal anti-inflammatory drugs (NSAIDs), may have a significant role. Pharmacokinetic studies conducted in elderly patients by Medellín-Garibay et al. [25] showed reduced clearance of amikacin when concomitant NSAIDs were administered. The described value of this parameter (2.75 L/h) differs greatly from values reported for other populations, e.g., 7.1 L/h found by Arechiga-Alvarado et al. [26] in younger patients, and 7.6 L/h in healthy volunteers (Garraffo et al.) [27]. The authors suggested that an extended dosing interval should be used in elderly overweight patients and/or those with lower creatinine clearance (<60 mL/min/1.73 m). The other commonly used nephrotoxic drugs including loop diuretics (e.g., furosemide or torsemide) or agents including cisplatin, amphotericin B, colistin, or vancomycin should therefore be minimized whenever feasible. In a multivariate logistic regression performed by Rybak et al., the concurrent use of vancomycin was a significant predictor of nephrotoxicity (*p* ≤ 0.001) [28]. There are no TDM-based data for the combined use of vancomycin and amikacin. The limited available evidence derives from studies with gentamicin, suggesting increased nephrotoxicity and the need for lower vancomycin exposure (AUC), with proposed targets below ~530 mg·h/L during combination therapy; however, these findings cannot be directly extrapolated to amikacin [29].

Nevertheless, in some clinical situations, the lack of safer alternatives may necessitate the unavoidable use of such combinations. The nephrotoxic effect of amikacin can be reversible if therapy is discontinued at an early stage. Clinical experience shows that early TDM can detect potentially nephrotoxic drug exposure at a stage when no clinical signs of acute kidney injury are yet apparent, allowing timely dose adjustment or drug discontinuation and thereby preventing the development of clinically manifest renal impairment. In multivariate analysis by Romdhane et al., only TDM-based dose adjustment remains a significant factor in the achievement of non-toxic trough levels [30]. More detailed discussion of the role of therapeutic drug monitoring is provided in Section 2.5 Therapeutic Drug Monitoring (TDM).

Moreover, ototoxicity represents a distinct and clinically significant adverse effect of amikacin, involving both cochlear and vestibular toxicity, due to its high affinity to these structures. Once inside, the drug is cleared very slowly. Its half-life in the inner ear fluid is significantly longer than in the blood, leading to a cumulative effect where damage continues to progress even after the drug is discontinued. Aminoglycosides appear to generate free radicals within the inner ear, with subsequent permanent damage to sensory cells and neurons. Vestibular toxicity typically manifests as dizziness and imbalance, whereas cochlear toxicity primarily affects high-frequency hearing and may initially be detectable only with audiometric testing. Aminoglycosides have variable cochleotoxicity and vestibulotoxicity. Amikacin, like neomycin and kanamicin, is primarily cochleotoxic, whereas streptomycin, tobramycin, and gentamicin are primarily vestibulotoxic [3,31]. Ototoxicity occurs both in a dose-dependent and idiosyncratic fashion. The idiosyncratic pathway is presumably due to genetic predispositions.

The genetic mechanism is linked to specific variants in the mitochondrial 12S rRNA gene (MT-RNR1), notably m.1555A > G and m.1494C > T [32]. The carrier frequency of m.1555A > G is approximately 0.1–0.5% in the general population, though there are sub-populations with higher prevalence. Documents from the UK NHS/CS Genomics cite a frequency on the order of ~1:500 (~0.2%) in the general population as an approximate value used when considering newborn screening [33]. This mutation causes the human mitochondrial 12S rRNA to resemble bacterial 16S rRNA, leading to aminoglycoside binding to mitochondrial ribosomes in the cochlear hair cells of the inner ear. The result is impairment of mitochondrial translation, respiratory chain dysfunction, increased oxidative stress, and ultimately apoptosis of the cochlear hair cells. These changes are usually not noticeable to the patient during therapy, but manifest after treatment as a sudden, often permanent hearing loss. In 2021, recommendations were published to consider genetic testing before planned long-term aminoglycoside therapy, provided it does not delay urgent treatment [34]. The current testing pathway is recommended for all age groups, and the guidance states that prophylaxis with oral N-acetylcysteine can be considered to prevent aminoglycoside-induced ototoxicity, although the evidence is limited [35].

In addition to nephrotoxicity and ototoxicity, aminoglycosides, including amikacin, may rarely induce neuromuscular blockade [3]. Although neurotoxicity has been observed mainly with local administration of aminoglycosides, such effects of amikacin can be relevant in patients receiving parenteral amikacin in combination with anesthetic agents or neuromuscular blocking drugs. Therefore, amikacin should be used with caution in patients with myasthenia gravis or other neuromuscular diseases such as Parkinson’s disease, because these drugs can exacerbate muscle weakness.

### 2.4. Amikacin Dosing Strategies

Currently, two dosing strategies are used: the traditional multiple daily dosing (MDD), which involves several doses per day, and the once daily dosing (ODD), which uses higher doses with extended dosing intervals (≥24 h). Rougier et al. [36], using pharmacokinetic modeling, suggested lower toxicity with ODD compared with MDD. However, this benefit did not persist during prolonged therapy. The study was limited by a small sample size (*n* = 35), relatively low daily doses (<700 mg), and exclusion of patients with baseline creatinine clearance <50 mL/min. Moreover, treatment efficacy and mortality outcomes were not assessed. Retrospective comparisons between dosing strategies should also be interpreted with caution due to potential bias. Despite the increasing use of ODD in clinical practice, compliance with guideline-recommended dosing remains suboptimal, with only 48% adherence reported by Namazi et al. [37]. These limitations become particularly relevant in patient populations with altered pharmacokinetics, where standard dosing strategies may be inadequate. Special populations, including critically ill patients (e.g., septic shock, augmented renal clearance, ECMO support, or severe burns) and pediatric and neonatal patients, often require individualized dosing. In obese patients, adjusted body weight should be used due to limited penetration of amikacin into adipose tissue.

This issue is further compounded by emerging evidence suggesting that standard dosing (15 mg/kg) may be insufficient to achieve pharmacodynamic targets in critically ill adults, particularly for pathogens with higher minimal inhibitory concentrations (MICs). Dia et al. [38] reported that a commonly used dose of 1500 mg achieves target exposure for MIC = 2 mg/L but is inadequate for MIC = 8 mg/L. Even higher doses (up to 3500 mg) may be insufficient in patients with high glomerular filtration rates (>96 mL/min/1.73 m^2^). Consistently, earlier studies reported that doses of 15–30 mg/kg often failed to achieve target peak concentrations [39,40,41,42]. Marsot et al. proposed doses up to 35 mg/kg for *Pseudomonas aeruginosa* infections in critically ill patients [43]. However, increasing doses may elevate the risk of acute kidney injury. Recent studies by Silva et al. [44], Yamamoto et al. [45] and Telles et al. [46] showed that the probability of target attainment increases with the grade of kidney impairment; however, estimated GFR did not predict amikacin elimination in critically ill patients with cancer. This underscores the necessity of therapeutic drug monitoring in critically ill patients with cancer.

The ODD strategy is supported by pharmacodynamic principles, including concentration-dependent killing and the post-antibiotic effect, and may reduce toxicity through saturable drug uptake in the renal cortex and inner ear. Prolonged periods of low serum concentrations may facilitate cellular drug clearance and limit toxicity. Taken together, these considerations underscore the central role of therapeutic drug monitoring (TDM) in optimizing both efficacy and safety of amikacin therapy.

### 2.5. Therapeutic Drug Monitoring (TDM)

Given the variability in pharmacokinetics and the limitations of fixed dosing strategies, TDM has emerged as a key tool for individualized therapy. Recent IDSA guidance (2024) recommends an initial dose of 15 mg/kg followed by therapy optimization based on TDM [6]. Monitoring of the area under the concentration–time curve (AUC), particularly the AUC/MIC ratio >8–10, is considered the preferred approach. Nomogram-based methods and trough concentration monitoring are regarded as alternatives. In clinical scenarios such as urinary tract infections, where high urinary concentrations are achieved, the added value of precise plasma AUC estimation for efficacy assessment may be limited. Furthermore, in empirical therapy, the application of AUC/MIC-based strategies is constrained by the lack of available MIC values. Evidence directly comparing nomogram-guided dosing versus trough-based adjustment in preventing nephrotoxicity remains limited, representing an important research gap.

Important limitations should be acknowledged in the studies cited by the IDSA as evidence base for amikacin dosing recommendations. Notably, the study by Drusano and Louie [47] addresses exclusively therapy with tobramycin and gentamicin and does not provide a separate analysis for amikacin. Extrapolation of pharmacokinetic and toxicological parameters from gentamicin to amikacin neglects structural differences between these molecules, which may affect tissue saturation kinetics and nephrotoxic potential. In the cited study, patients were treated predominantly for pneumonia, a clinical context in which aminoglycoside monotherapy is currently considered inappropriate and is not recommended by any major scientific society. The methodology used to assess treatment efficacy also raises substantial concerns. The primary efficacy endpoint was defined as “time to defervescence”, a soft surrogate with limited predictive value for clinical outcomes or mortality. Moreover, the information on concomitant use of antipyretics, corticosteroids or other interventions influencing fever was not provided. The same endpoint has also been employed in other studies. Furthermore, with respect to the statistical reliability of the safety analysis, no cases of nephrotoxicity were observed in the ODD group, which substantially limits the robustness of the safety assessment. The use of historical creatinine-based definitions of renal injury rather than contemporary criteria may have further contributed to the underestimation of acute kidney injury risk. It should also be noted that the MIC values reported at the time of publication are no longer representative in the current era of widespread antimicrobial resistance, including resistance mechanisms such as carbapenemases. These limitations underscore the need for real-world comparative studies evaluating different TDM strategies.

In this context, target concentrations used in therapeutic drug monitoring warrant closer examination. Target concentrations, including trough levels, are defined separately for different dosing regimens. The most conservative recommendations are provided by the Sanford Guide (summarized in Table 1), which suggests maintaining trough concentrations below 1 mg/L [48]. Both the IDSA and Polish national guidelines for amikacin recommend a trough concentration below 5 mg/L; however, this threshold is supported by only a limited number of small studies. A meta-analysis including a total of 159 patients demonstrated that amikacin trough concentrations below 10 mg/L were associated with a significantly lower risk of nephrotoxicity compared with higher levels [49]. Notably, intermediate thresholds, such as trough concentrations below 2.5 mg/L, have also been reported and may represent a pragmatic target in clinical practice, given the availability of current analytical methods [50,51]. A systematic review by Jenkins et al. emphasized that, despite the available studies, an optimal dosing regimen and clearly defined TDM targets for amikacin have not been established, and current practice remains largely guided by expert consensus rather than high-quality evidence [52].

In critically ill patients, antimicrobial TDM increasingly relies on model-informed precision dosing (MIPD), integrating population pharmacokinetic models with Bayesian forecasting to account for profound pharmacokinetic variability. Among the studies included in this review, the majority were conducted in critically ill populations, particularly oncology patients, and numerous pharmacokinetic models—predominantly one- and two-compartment—have been developed to support individualized dosing. These approaches are reflected in international recommendations, including the position paper by Abdul-Aziz et al. (2020), which endorses routine TDM for aminoglycosides in ICU settings [53]. Additional data, including studies in patients receiving extracorporeal membrane oxygenation (ECMO) [54] and the AMIDIAL-ICU study [23] in patients undergoing renal replacement therapy, further support the use of advanced pharmacokinetic modeling in highly dynamic clinical conditions. Overall, MIPD-based TDM in critically ill patients is well justified and supported by a growing body of evidence, despite ongoing practical limitations related to implementation.

In contrast, data on TDM in non-critically ill patients remain limited, despite this population representing the majority of hospitalized individuals. Only a few studies were identified. Kim et al. stated that the probability of occurrence of nephrotoxicity in noncritically ill patients is less known and tends to be overestimated [55].

Goncalves et al., in a study of 628 patients, reported that peak concentrations remain relatively stable, whereas trough levels vary substantially, supporting the clinical utility of trough-based assessment, particularly in elderly patients [56]. Such an approach may represent a practical alternative in settings with limited access to clinical pharmacy expertise, as it does not require advanced modeling or intensive sampling.

Another study, performed by Jayakumar [57] on a group of 125, concluded that monitoring through level can be used to predict the occurrence of nephrotoxicity.

Moreover, minimizing the number of blood sampling procedures may reduce procedure-related risks and alleviate demands on healthcare personnel. Kovacevic et al. questioned the utility of TDM in this context; however, their conclusions are limited by small sample size, use of lower dosing regimens, and non-contemporary dosing strategies, while other authors support routine TDM for all amikacin-treated patients [58].

TDM is mainly and optimally performed by pharmacists supported by appropriate software [59]. Alhameed et al. [60] and Dvořáčková et al. [61] suggested that computer-assisted therapeutic drug monitoring of aminoglycosides by pharmacists leads to better safety therapeutic outcomes and cost avoidance. However, no statistically significant differences were found between the groups based on the physician’s experience.

The role of nomogram-based monitoring remains insufficiently defined despite its inclusion in IDSA recommendations. In one of the analyzed studies, a single concentration measured 10 h after dosing was used for nomogram-based dose adjustment [62]. Based on the above analysis, it may seem justified to choose the TDM method depending on the population, which is presented in Table 2.

## 3. Discussion

One of the key gaps identified in the literature is the lack of robust data on the true incidence of amikacin-induced toxicity and on the comparative effectiveness of different TDM strategies in reducing the risk of acute kidney injury. Current guidelines vary in their recommended target trough levels for safety (with the IDSA and Polish national guidelines suggesting <5 mg/L, versus the more conservative threshold of <1 mg/L proposed by the Sanford Guide), yet these thresholds are based on limited evidence. The only recent meta-analysis published by Yamada et al. [49], including 159 patients, demonstrated that concentrations below 10 mg/L were associated with significantly lower risk of nephrotoxicity compared with higher levels.

Particular uncertainty exists in patients with intermediate trough concentrations, for whom clear evidence-based guidance is lacking. Currently, the range of measured trough concentrations between 1 and 5 µg/mL represents a poorly defined range, largely left to individual clinical judgment. Patients within this range may be at a higher risk of nephrotoxicity compared with the general population, making them potential candidates for intensified therapeutic drug monitoring, for example through model-informed precision dosing (MIPD) and Bayesian-based TDM approaches.

No head-to-head studies have directly compared MIPD-dosing, nomogram-guided dosing and trough-concentration monitoring in terms of nephrotoxicity prevention.

Importantly, the available evidence is heavily skewed toward critically ill populations, in whom pharmacokinetic variability is most pronounced and where model-informed precision dosing (MIPD) approaches are increasingly supported. In contrast, data on non-critically ill patients remain scarce, despite this group representing the majority of hospitalized individuals. In these patients, available studies suggest that trough concentrations may provide a pragmatic basis for therapeutic drug monitoring, as peak concentrations tend to remain relatively stable while trough levels vary more substantially in this group. This raises the question of whether simplified TDM strategies may be sufficient in selected clinical settings, particularly where access to advanced pharmacometric tools is limited.

Taken together, these findings suggest that while advanced, model-based TDM strategies are well justified in critically ill patients, their routine application in non-critically ill populations may not always be necessary and should be balanced against feasibility, resource availability, and clinical context.

Beyond acute kidney injury, ototoxicity represents another clinically relevant adverse effect of amikacin, the risk of which is strongly influenced by genetic susceptibility. An increasing body of evidence indicates that variants in the mitochondrial 12S rRNA gene (most notably m.1555A > G and m.1494C > T) markedly increase the risk of aminoglycoside-associated hearing loss, which may occur at therapeutic concentrations even after short-term exposure and may not be fully preventable by TDM alone; genetic testing has therefore been proposed as a potential strategy to improve treatment safety in selected clinical settings.

Our critical appraisal of current guidelines highlights that some recommendations for amikacin dosing (such as AUC/MIC targets in urinary tract infections) may not be optimally evidence-based, given amikacin’s extremely high urine concentration. This underscores the importance of considering the clinical context when selecting TDM strategies, as more complex approaches may increase healthcare costs and expose patients to additional procedures with potential complications.

In addition, patient-related factors substantially influence the pharmacokinetics and safety profile of amikacin. Special populations may often require individualized dosing and monitoring strategies. In these settings, standard dosing regimens may be insufficient to achieve therapeutic targets for less susceptible organisms, prompting consideration of higher or more frequent dosing, albeit with careful attention to the associated increase in toxicity risk. Co-administration of amikacin with nephrotoxic agents, particularly vancomycin, has been consistently associated with an increased risk of kidney injury and should therefore be avoided whenever clinically feasible.

Taken together, these considerations highlight that the safe and effective use of amikacin cannot rely on uniform dosing or monitoring paradigms. Instead, context-specific, patient-tailored strategies integrating pharmacokinetic principles, careful therapeutic monitoring, and avoidance of modifiable risk factors are required. Further prospective studies are needed to directly compare therapeutic drug monitoring approaches in real-world settings and to refine dosing and monitoring strategies across distinct patient populations.

## 4. Materials and Methods

A literature search in Medline (via PubMed) and Embase was performed to identify clinical studies on amikacin pharmacotherapy with particular focus on dosing strategies, TDM and treatment safety (including nephrotoxicity and ototoxicity). The search was conducted from January 2015 to December 2025 and was limited to studies in humans and publications in the English language. Controlled vocabulary terms (Medical Subject Headings and Emtree terms) were combined with free text keywords related to amikacin, TDM, pharmacokinetics, dosing strategies, and adverse drug reactions. The studies on Mycobacterium infections (e.g., tuberculosis) were excluded by appending NOT filters in the query. This exclusion was important because amikacin is often administered via inhalation in mycobacterial infection studies, leading to distinct pharmacokinetics and toxicity profiles that could introduce heterogeneity into our analysis. The PubMed search string included:

(((((“amikacin/administration and dosage”[MeSH Terms] OR “amikacin/adverse effects”[MeSH Terms] OR “amikacin/therapeutic use”[MeSH Terms] OR “amikacin/toxicity”[MeSH Terms] OR (“amikacin/adverse effects”[MeSH Major Topic] OR “amikacin/administration and dosage”[MeSH Major Topic] OR “amikacin/toxicity”[MeSH Major Topic] OR “amikacin/therapeutic use”[MeSH Major Topic] OR “amikacin/toxicity”[MeSH Major Topic])) AND (“humans”[MeSH Terms] AND 2015/01/01:2025/12/31[Date—Publication]) AND “drug monitoring”[MeSH Terms]) NOT “neonat*”[All Fields]) NOT “tuberculosi*”[All Fields]) NOT “mycobacter*”[All Fields]) AND (“Adult”[MeSH Terms] OR “Aged”[MeSH Terms]) AND (english[Filter]).

**Figure 1:**
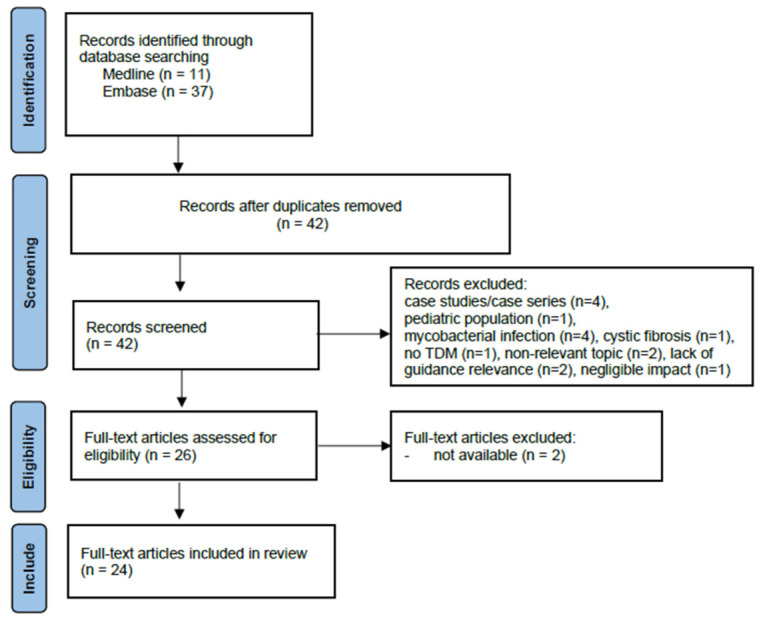
Flow diagram illustrating the study selection process.

The PubMed query yielded 11 records capturing the majority of clinically relevant original studies and reviews on amikacin TDM and safety included in this narrative review. An analogous search strategy was executed in Embase using the corresponding Emtree terms and keywords, combined in the same manner. The corresponding Embase search strategy was constructed using analogous Emtree terms and keywords and applied with equivalent limits. The search string was as follows:

‘drug monitoring’ AND amikacin:ti AND (2015:py OR 2016:py OR 2017:py OR 2018:py OR 2019:py OR 2020:py OR 2021:py OR 2022:py OR 2023:py OR 2024:py OR 2025:py) AND [english]/lim AND [humans]/lim NOT (tuberculosi*.ti. OR mycobacter*.ti.) AND (‘adult’:ag OR ‘aged’:ag OR ‘middle aged’:ag OR ‘very elderly’:ag OR ‘young adult’:ag), yielding a total of 37 articles. After running the searches, we merged the results from PubMed and Embase and removed duplicates. The overall study selection process is summarized in a flow diagram (Figure 1), adapted from PRISMA principles, illustrating the identification, screening, and inclusion of studies (*n* = 24).

In order to provide a comprehensive pharmacological and clinical context, selected key publications addressing fundamental pharmacokinetic principles, tissue penetration, antimicrobial spectrum and guideline recommendations used in daily clinical practice were additionally included, even if they did not emerge directly from the database search strategy. This manual search included foundational sources published prior to 2016 (*n* = 15), as well as other relevant clinical guidelines and key papers (*n* = 17).

Summing up, in this narrative review, a total of 56 articles were discussed: 24 were identified through a systematic search of two databases, while 32 additional articles were purposively selected based on their clinical significance and the authors’ professional experience.

## 5. Conclusions

Amikacin remains an important option in the therapy of severe and complicated infections. However, its clinical use is constrained by the risk of nephrotoxicity and ototoxicity. Available evidence supports the preferential use of ODD, which demonstrates a safety profile at least comparable to, and often more favorable than, MDD while facilitating attainment of pharmacodynamic targets in many clinical settings.

Safe use of amikacin requires integrated risk mitigation that includes TDM and, where applicable, genetic testing. The primary objective of monitoring varies according to the clinical context, reflecting differences between indications and patient populations.

Although current guideline recommendations regarding target trough concentrations are inconsistent and supported by limited evidence, they provide a practical framework for clinical decision-making.

Further prospective studies are warranted to strengthen the evidence base, directly compare simplified monitoring strategies, and refine dosing and monitoring approaches to optimize the clinical use of amikacin.

## Figures and Tables

**Table 1:** Recommended concentrations for amikacin according to dosing regimen (adapted from the Sanford Guide).

	ODD (1 × 15 mg/kg)	MDD (2 × 7.5 mg/kg)
Cmax	56–64 mcg/mL	15–30 mcg/mL
Cmin	<1 mcg/mL	5–10 mcg/mL

Abbreviations: ODD, once-daily dosing; MDD, multiple daily dosing; Cmax, peak concentration; Cmin, trough concentration.

**Table 2:** Proposed therapeutic drug monitoring strategies for amikacin in different patient populations.

Patient Populations	Recommended TDM Approach	Rationale
Critically ill cancer patients, ECMO, RRT	MIPD, Bayesian models	Very high variability, rapidly changing clearance
Non-critically ill with limited factors of nephrotoxicity or limited resource settings	Through monitoring, nomogram	Stable peak concentrations, variability of through levels Limited access to pharmaceutical expertise, feasibility [6,56,57]

Abbreviations: ECMO, extracorporeal membrane oxygenation; RRT, renal replacement therapy; MIPD, model-informed precision dosing.

## Data Availability

No new data were created or analyzed in this study. Data sharing is not applicable to this article.

## Abbreviations

The following abbreviations are used in this manuscript:

AUC	area-under the curve
CDI	*Clostridioides difficile* infection
ECMO	extracorporeal membrane oxygenation
ELF	epithelial lining fluid
ICU	intensive care unit
IDSA	Infectious Diseases Society of America
MDD	multiple daily dosing
MICs	minimal inhibitory concentrations
MIPD	model-informed precision dosing
NSAIDs	non-steroidal anti-inflammatory drugs
ODD	once-daily dosing
PAE	post-antibiotic effect
RRT	renal replacement therapy
TDM	therapeutic drug monitoring
UTI	urinary tract infections
VAP	ventilator-associated pneumonia
